# Cross-cultural and international barriers and enablers to medication safety and pharmacotherapy: insights from a World Café study on interprofessional education

**DOI:** 10.1007/s00228-026-04128-1

**Published:** 2026-07-10

**Authors:** Rowan Sultan, Jan-Jaap Reinders, Michael O. Reumerman, Michiel A. van Agtmael, Milan C. Richir, Jelle Tichelaar

**Affiliations:** 1 Department of Internal Medicine Unit Pharmacotherapy, Amsterdam UMC, Vrije Universiteit, De Boelelaan 1118, Room ZH-4A50, 1081 HZ Amsterdam, The Netherlands; 2 Research and Expertise Center in Pharmacotherapy Education (RECIPE), Amsterdam, The Netherlands; 3 Interprofessional Collaboration and Medication Safety at the Faculty of Health, Sports and Social Work, Inholland University of Applied Sciences, Amsterdam, The Netherlands; 4 Research Group Interprofessional Education (IPE), LEARN, University Medical Center Groningen, University of Groningen, Groningen, The Netherlands; 5 Research Group Healthy Ageing, Allied Health Care and Nursing, Hanze University of Applied Sciences, Groningen, The Netherlands; 6 Research Domain Interprofessional Identity and Collaboration, Research Line Oral Health for Life, Center of Dentistry and Dental Hygiene, University Medical Center Groningen, University of Groningen, Groningen, The Netherlands; 7 Department of Surgery, University Medical Center Utrecht, Utrecht, The Netherlands

**Keywords:** World Café, Interprofessional education, Interprofessional education and collaborative practice, IPE, IPECP, Interprofessional identity, Clinical pharmacology, Medication safety, Medical education, Pharmacotherapy

## Abstract

**Introduction:**

The prevalence of chronic illnesses and polypharmacy has made medication safety complex. While interprofessional collaboration has been proposed as a strategy to improve medication safety and pharmacotherapy, little is known about the barriers and enablers that influence its implementation in this context. This study aims to identify these barriers and enablers, using the Interprofessional Education for Collaborative Patient-Centred Practice (IECPCP) framework.

**Methods:**

A World Café study was carried out at the All Together Better Health XI conference in Doha, Qatar, in November 2023. Participants engaged in discussions on four key themes related to interprofessional education in pharmacotherapy and medication safety. Data were analyzed using the IECPCP framework.

**Results:**

Seventeen participants from seven countries identified 41 barriers and 75 enablers. Thematic analysis revealed six overarching domains: Culture and Attitude, Policy and Governance, Structure and Curriculum, Roles and Identity, Resources and Support, and Evaluation and Evidence. Key barriers included hierarchical norms and a lack of clarity about interprofessional roles at macro and meso levels, and interpersonal conflict and limited accountability at a micro level. Enablers included the promotion of open communication, leadership support, integration of interprofessional education into curricula, and the use of real-world examples to improve the relevance of training.

**Conclusion:**

National laws, healthcare policies, and cultural hierarchies hinder interprofessional education in pharmacotherapy and medication safety, and strict legal rules and professional responsibilities concerning prescribing complicate collaboration. Stimulating open communication between different professionals may help improve interprofessional collaboration and create more supportive conditions for safe prescribing and medication safety.

**Supplementary Information:**

The online version contains supplementary material available at 10.1007/s00228-026-04128-1.

## Introduction

The global population is aging and the prevalence of chronic illnesses is increasing [1], such that there has been a marked increase in patients suffering from multiple chronic conditions, leading to an increased prevalence of polypharmacy, the concurrent use of five or more medications [1]. At the same time, healthcare delivery is changing, with more and diverse healthcare providers being licensed to prescribe and monitor medications [2]. This, together with polypharmacy, makes medication safety increasingly challenging. In this context, interprofessional collaboration is increasingly recognized as a key strategy to support safe prescribing, medication safety, and ultimately patient outcomes [3–5].

Studies show that current healthcare providers are insufficiently capable of collaborating interprofessionally [6, 7]. This is not surprising given that most educational curricula are designed in a monoprofessional manner [8, 9]. To better prepare the prescribers of the future for interprofessional collaboration, a growing number of educational interventions have been developed to promote collaboration among undergraduate students from different professions [10–12]. This contributes to the development of an interprofessional identity and a change in hierarchical communication between students, which in turn leads to less hierarchical communication upon graduation as healthcare providers [13–17]. Moreover, the development of an interprofessional identity increases the likelihood of collaboration with other healthcare professionals [16, 18–21].

Multiple studies have explored the barriers and enablers to developing interprofessional educational interventions [22, 23], yet none have specifically examined these factors in the context of pharmacotherapy and polypharmacy, where medication errors are highly prevalent, largely preventable, and can cause significant morbidity and mortality [24, 25]. Understanding barriers and enablers requires paying attention to the broader cultural and contextual conditions that shape interprofessional collaboration, based on the subjective interpretation of environmental factors, which is influenced by cultural values, norms, the local environment, and local infrastructure. Social identities, including professional identities, are shaped by these contextual elements and triggered by specific cues, or identity triggers. An identity trigger is a contextual cue that heightens the salience of a social or personal identity and shapes related responses [26–29]. The same is true for interprofessional identity which, when well-developed, motivates professionals to collaborate in ways that prioritize shared goals and coordinated practice instead of simply dividing or delegating tasks [20]. Professional and interprofessional identity triggers, often learned through socialization, can prompt identity-driven professional action as well as interprofessional collaboration. Thus, cultural and contextual factors can either facilitate or hinder interprofessional collaboration in prescribing, and comparison of these factors could help identify universal factors and mechanisms that support effective interprofessional collaboration in this high-risk area. In 2017, the WHO launched the Third Global Patient Safety Challenge: Medication Without Harm with the aim of improving medication safety. The associated reports identify the importance of interprofessional collaboration in achieving this goal, given that challenges regarding medication safety exist at every stage of medication use [30].

Oandasan and D’Amour proposed a model that highlights micro-, meso-, and macro-level factors influencing interprofessional collaboration [27], In this study, we used their model to identify international barriers and enablers for the implementation of interprofessional education in the context of pharmacotherapy and medication safety.

## Methods

This World Café was organized at the All Together Better Health XI (ATBH XI) conference, an international conference held in Doha, Qatar in November 2023. The central theme of the conference was interprofessional education and collaborative practice (IPECP) and gave participants the opportunity to exchange ideas and best practices (ATBH XI | Interprofessional.Global). The international conference setting provided an opportunity to explore perspectives on interprofessional education in pharmacotherapy and medication safety across different healthcare and educational contexts.

### Study design

**Fig. 1:**
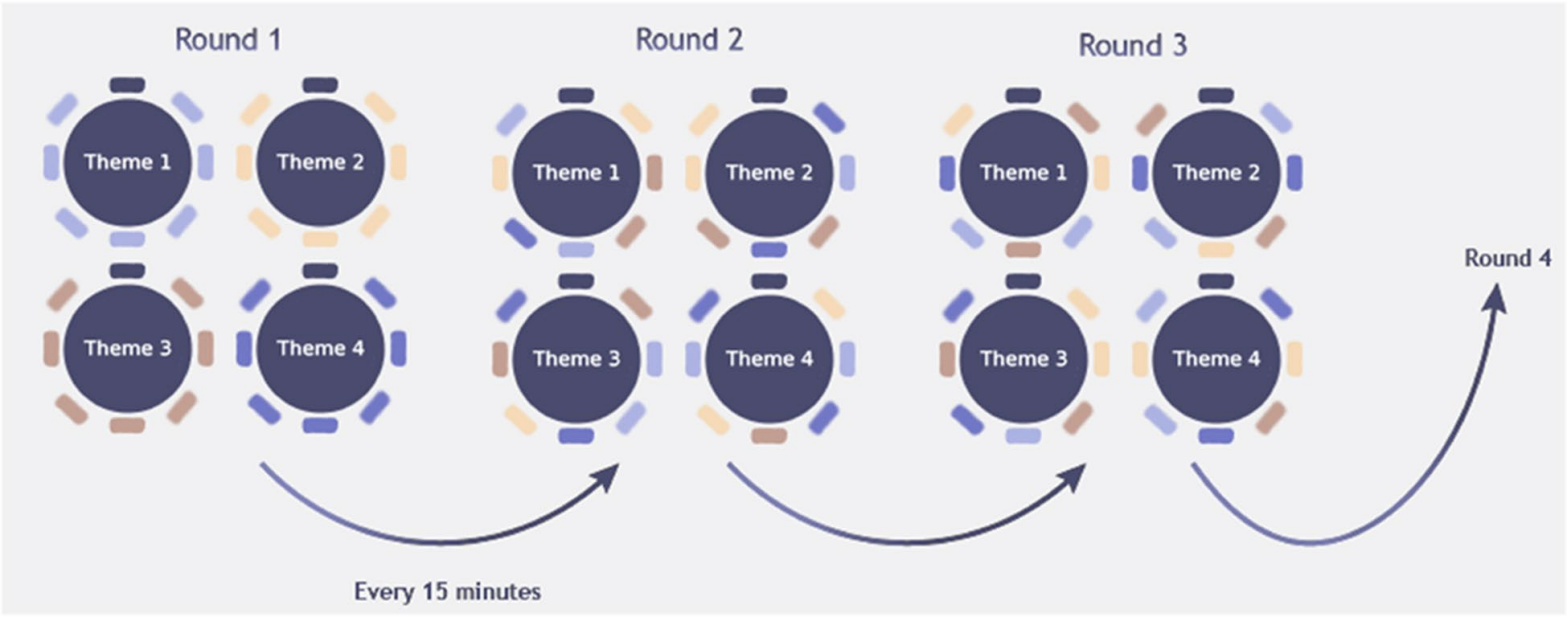
Structure of the World Café sessions. Participants discussed four different themes in successive rounds of 15 min. After each round, participants rotated to a new table (“cross-pollination”), allowing them to build on ideas from previous groups. In the final round, all participants had contributed to each theme

We used the World Café method because of its effectiveness in stimulating innovative thinking among individuals meeting for the first time and facilitating discussions in large groups. World Café sessions typically consist of several rounds of discussions focused on different themes, during which participants rotate among tables. The following seven Café principles were followed to increase the likelihood of active engagement, authentic dialogue, and constructive possibilities for action [31]:

1. *Set the context*
  One of the researchers (R.S.) started with a short presentation, informing the participants about the World Café, about the general rules, and about the study.
2. *Create hospitable space*
  Before the start of the World Café, everyone in the room introduced themselves in order to get to know each other before engaging in the discussions. The room was set up with four tables and four chairs each. A moderator (R.S., J.J.R., M.O.R., J.T.) sat at each table and documented the discussion on a flip chart using markers, while participants wrote their ideas on post-it notes.
3. *Explore questions that matter*
  Prior to the conference, the research team identified four key themes regarding interprofessional education in the field of medication safety. Each table discussed one of these themes: 1. Implementation: Interprofessional education in medication safety should be offered as a standard component in all healthcare curricula; 2. Logistics: Optimized logistics in interprofessional education in medication safety is essential to ensure that participants engage in meaningful experiences; 3. Urgency: Interprofessional education in medication safety is of great importance in preparing healthcare providers for the dynamic healthcare landscape; 4. Study outcomes: Study outcomes of initiatives in interprofessional medication safety education should focus on healthcare outcomes instead of learning outcomes.
  Participants were repeatedly encouraged to consider these statements from the viewpoint of pharmacotherapy, so that all discussions focused on safe and effective prescribing, which is central to medication safety in interprofessional education.
4. *Encourage everyone’s contribution*
  Each session lasted 15 minutes, and each table had four participants. In the first 2–3 minutes the participants were asked to write down potential barriers and enablers for that table’s theme on the post-it notes, after which the participants could share and discuss their thoughts. Moderators guided the discussion and ensured that all participants had an opportunity to contribute.
5. *Cross-pollinate and connect diverse perspective*
  After 15 minutes the participants switched tables at random (cross-pollination), which was repeated until all participants had discussed the four themes (Fig. 1). During rounds 2, 3 & 4, the moderator (who remained seated at the same table each round) summarized the information mentioned during the previous rounds, and the participants built on this information, suggesting new barriers and enablers. The moderators observed that by the end of the final round, recurring themes were repeating with minimal new insights emerging, indicating that thematic sufficiency had been achieved.
6. *Listen together for patterns, insights and deeper questions*
  The moderators asked participants probing questions to further investigate the barriers and enablers mentioned. Flip charts were used to record the barriers and enables identified by the participants, which facilitated the recognition of patterns and the linking of barriers to solutions.
7. *Harvest and share collective discoveries*
  After completion of all four rounds, the moderator reported the most important aspects that had been discussed at his/her table. All participants then had the chance to comment on this. No audio or visual recordings were made, as the World Café method prioritizes real-time, co-created data capture through post-it notes and flip charts, and the dynamic, multi-table discussions limit the feasibility and added value of audio recording. To enhance data completeness and credibility, moderators actively summarized discussions during and after each round, and participants were given the opportunity to review and validate the recorded notes.

### Ethical considerations

This project was approved by the Medical Ethics Review Committee of Amsterdam UMC, location VU medical centre (2023.0644).

### Participants

All attendees of the ATBH XI conference were invited to participate in the workshop and this study through the official conference program and on-site announcements prior to the session. Participation was based on convenience sampling, as attendees could voluntarily join the workshop. Interested participants were approached face-to-face at the start of the session and provided with study information before giving written informed consent. Participants were working in various medical fields (e.g. medicine, pharmacy, dentistry, speech therapy) and shared a common interest in interprofessional education in medication safety (Table 1).

**Table 1:** Participant characteristics of participants included in the World Café study, including gender distribution and country of origin

Characteristic	*N*
Total participants	17
Gender	
Female	9
Male	8
Country	
Qatar	7
The Netherlands	4
United States of America	2
Turkey	1
Singapore	1
Canada	1
Thailand	1

### Data collection and analysis

The post-it notes and flip charts were collected for data analysis. The barriers and solutions were collated for each theme and analyzed using the Framework Method [32]. Initial coding was performed independently by two researchers (R.S. and J.T.), after which codes were compared and discussed within the research team to reach consensus. In this study, we used the Interprofessional Education for Patient Centred Practice (IECPCP) framework of Oandasan and D’Amour, which categorizes factors influencing interprofessional education into macro, meso, and micro levels [33]. The micro level focuses on individual learners and educators, including their skills, attitudes, and the direct teaching methods used. The meso level covers the organizational environment, such as available resources, leadership support, and curriculum design. The macro level encompasses the broader system and policy context, including laws, regulations, funding, professional boundaries, and wider hierarchical norms embedded in national healthcare traditions and professional structures [33]. We then carried a thematic analysis to inductively identify overarching themes [34]. Barriers and enablers identified from post-it notes and flip charts were first coded and grouped into subthemes based on conceptual similarity in Microsoft Excel. These subthemes were subsequently organized into six overarching thematic domains. Finally, themes were mapped onto the macro-, meso-, and micro-levels of the IECPCP framework, resulting in the structured presentation of findings in Supplementary Tables S1–S3. The tables therefore represent the final stage of the analytical process, linking raw data to thematic interpretation. In line with the framework’s emphasis on evaluation, we also included outcome-related statements to highlight the role of research in strengthening the implementation of interprofessional education.

**Table 2:** Summary of the key barriers and corresponding enablers for interprofessional education in pharmacotherapy and medication safety across the macro-, meso-, and micro-levels Detailed barriers and enablers are provided in Supplementary Tables S1–S3

Level of barrier	Key barriers	Corresponding enabler
Macro	Cultural hierarchies hinder interprofessional collaboration	Integration of IPE into accreditation standards and national policies
	Professional overconfidence hinders interprofessional collaboration	Structured interprofessional discussions on medication safety to challenge professional overconfidence
Meso	Rigid curricula and limited institutional support	Embedding IPE within authentic clinical activities and existing curricula
	Strong resistance to change slows the adoption of interprofessional practices	Providing evidence that interprofessional education improves patient safety in practice
Micro	Strong professional identity can cause discomfort or shame when stepping outside traditional roles	Building awareness of professional identity as part of collaboration

The Consolidated Criteria for Reporting Qualitative Research (COREQ) checklist for reporting interviews and focus groups was used in this study [35].

### Research team

As described by Giorgi, thematic analysis requires an open mind to allow unexpected meanings to emerge [36]. However, researchers always bring their own assumptions and beliefs. The four moderators in this World Café were R.S., J.J.R., M.O.R, and JT. Three were male (J.J.R., M.O.R., J.T.) and one was female (R.S.). M.O.R. and J.T. are clinical pharmacologists, R.S. is a clinical pharmacologist in training, and J.J.R. is an organizational psychologist. All except R.S. hold a doctorate degree. R.S. conducted this research as part of her PhD project. The moderators were experienced in qualitative research methods, including focus groups, interviews, and World Café sessions. The research team had no prior relationships with the participants. No financial or professional incentives created a conflict of interest.

## Results

Seventeen participants from seven countries (Qatar, The Netherlands, USA, Turkey, Singapore, Canada, Thailand), all interested in interprofessional education concerning medication safety, were involved: 8 medical doctors, 2 pharmacists, 1 speech therapist, 1 psychologist, 3 medical students, and 2 dental students. No participants withdrew after providing consent. Participant characteristics are summarized in Table 1.

### General findings

After four iterative rounds of 15-min, thematic sufficiency was achieved. A total of 41 barriers and 75 enablers were identified. One or more enablers were described for 38 of the barriers; no enablers were reported for the remaining 3 barriers. Thematic analysis identified six overarching domains: Culture and Attitude; Policy and Governance; Structure and Curriculum; Roles and Identity; Resources and Support; and Evaluation and Evidence. To avoid repetition, the results are presented according to these domains, with each theme including insights across all levels of implementation. A summary of the key findings is presented in Table 2; detailed barriers and corresponding enablers are available in Supplementary Tables S1–S3.

### Culture and attitude

Cultural factors were identified as barriers to interprofessional education across all levels. At the macro level, these included over-reliance on individual expertise, hierarchical norms, and a false sense of safety around medication practices, which constrained collaboration. Promoting transparency through open safety discussions and embedding interprofessional education in accreditation standards were suggested to challenge these norms.

At the meso level, institutional resistance to change emerged as a key issue, with real patient safety incidents, visionary leadership, and committed facilitators identified as drivers for sustainable cultural change.

At the micro level, interpersonal conflict and limited accountability within interprofessional teams were seen as major obstacles. Creating psychologically safe learning environments, supporting open communication, and emphasizing the unique contributions of each profession to patient safety were proposed to address these challenges.

### Policy and governance

At the macro level, a lack of perceived urgency among policymakers was frequently identified. Framing interprofessional education as a healthcare priority and using international examples were suggested to strengthen policy support.

At the meso level, competing organizational priorities and limited institutional support hindered sustainable implementation. Developing structured guidelines and strengthening leadership were proposed to improve continuity of practice.

At the micro level, sudden curricular changes and the absence of structured implementation strategies disrupted long-term initiatives, highlighting the need for continuity and long-term planningto safeguard the sustainability of efforts to provide interprofessional education.

### Structure and curriculum

Curricular barriers were most evident at the meso and micro levels. At the meso level, rigid curricula, misaligned timetables, and limited integration across disciplines hindered implementation. Suggested solutions included embedding interprofessional education in core curricula, providing protected time, and using realistic, case-based learning.

At the micro level, uncertainty around defining and assessing competencies, as well as concerns about authenticity, were key issues. Integrating interprofessional education into existing formats such as problem-based or team-based learning and using real-world scenarios were proposed to increase relevance.

### Roles and identity

At the meso level, professional identity both constrained and enabled interprofessional collaboration. Role ambiguity and professional ego limited cooperation, while training focused on clarifying roles and the use of interprofessional role models were identified as key enablers.

At the micro level, students experienced discomfort when moving beyond their own professional boundaries. Strengthening role awareness, peer-led teaching, and team-based reflection were suggested to support collaboration.

### Resources and support

Resource constraints were identified across all levels. At the macro level, high implementation costs were a key barrier, with cost-effectiveness studies proposed to support funding decisions and policy development.

At the meso level, limited funding, staff shortages, and insufficient infrastructure hindered implementation. Suggested solutions included financial support, scalable models, and training additional facilitators.

At the micro level, limited time and a lack of expert educators were reported. Faculty development, multidisciplinary teaching teams, and integration into existing schedules were proposed to address these challenges.

### Evaluation and evidence

Limited evidence for the effectiveness of interprofessional education was identified as a key challenge, particularly at the macro and meso levels. At the macro level, difficulties in measuring long-term outcomes and attributing effects were major barriers. Suggested solutions included defining shared outcome measures and focusing on shorter-term or retrospective evaluations.

At the meso level, insufficient understanding of topics such as polypharmacy reduced perceived relevance. Using clinical examples and real-world data on medication errors was proposed to enhance engagement.

At the micro level, few barriers were directly related to evaluation, although integrating assessment into interprofessional education activities was considered important to reinforce legitimacy.

## Discussion

Using the IECPCP framework of Oandasan and D’Amour [33], we identified macro-, meso-, and micro-level barriers and enablers affecting medication safety and pharmacotherapy, with particular attention to the role of interprofessional education and collaboration. We found that practical barriers, such as difficulties in scheduling, limited experience with interprofessional education, and unclear professional roles, were largely similar across different educational domains, such as clinical reasoning and diagnostics. However, the real differences emerged at the macro level. This level includes national laws surrounding medication safety, healthcare policies, and cultural hierarchies. While many of the identified barriers align with broader interprofessional education literature, our findings suggest that hierarchical dynamics may be more pronounced in the context of pharmacotherapy. This is likely related to the strong link between prescribing authority, professional responsibility, and patient safety, which can reinforce traditional hierarchies and limit shared accountability [37].

As a result, existing interprofessional education challenges may require additional attention when applied to pharmacotherapy and medication safety. This suggests that applying general interprofessional education principles to pharmacotherapy may require more explicit consideration of prescribing-related hierarchies [30, 38]. Unlike competencies such as clinical reasoning or diagnostics, where tasks can be shared more freely across professions, prescribing is legally restricted to licensed professions and is strongly influenced by professional regulations and healthcare policies [39]. This creates unique challenges for IPE, as students who will eventually become prescribers must collaborate with students from other healthcare professions who will not have prescribing authority but can still contribute valuable input to medication plans. Effective IPE in this context requires awareness of one’s own professional role without reinforcing hierarchy and promotes clear and respectful communication. In addition, it should support the development of both professional and interprofessional identity and include debriefing sessions that explicitly address role boundaries, collaborative practice, and hierarchical dynamics [37, 40].

To the best of our knowledge, this is the first study to investigate interprofessional education regarding pharmacotherapy and medication safety. Our approach allowed us to uncover specific themes, such as the need to address polypharmacy, clarify prescribing accountability, and promote team-based medication management, themes which are often overlooked in more general interprofessional education discussions.

Our findings align with earlier research showing that the biggest challenges for interprofessional education in pharmacotherapy and medication safety occur at the national and system level and include strict prescribing laws, rules that restrict what different professions are allowed to do, and healthcare systems that are complicated and fragmented [41, 42]. For example, in the Netherlands and Sweden, unclear regulations and separate curricula make it difficult to align interprofessional education on medication safety [40]. In Qatar, strong hierarchies in medicine and limited policy support hinder collaboration [43, 44]. In low-income countries, low funding, too few trained teachers, and weak policy frameworks further add to the difficulties [45].

In contrast, at the meso and micro levels, barriers such as institutional resistance, limited time, financial support, and professional silos are consistent across all interprofessional education areas including pharmacotherapy, communication, and diagnostics [22].

### Strengths and limitations

A key strength of this study is the diverse mix of participants, both internationally and professionally. They represented a range of cultural, professional, and educational backgrounds. The inclusion of both students and educators, especially those with experience in interprofessional education and pharmacotherapy, supported a broad range of perspectives during the discussions. The open and respectful atmosphere, combined with a clear structure and the use of the World Café method, encouraged everyone to contribute [31, 46]. The World Café is recognized as a suitable methodology to structure discussions on complex topics in a way that ensures inclusivity and equal participation, allowing all participants, including those less outspoken, to be heard and acknowledged. This study used a clear framework to explore the barriers and enablers of interprofessional education in pharmacotherapy and medication safety. By looking at factors at macro, meso, and micro levels, we gained a comprehensive and cross-cultural picture of how policies, institutions, and team dynamics affect interprofessional education in medication safety. The use of the IECPCP framework of Oandasan and D’Amour to structure our analysis strengthened this approach, as it offers a validated way to examine these levels. This made it easier to compare our findings with other areas of interprofessional education, showing where pharmacotherapy faces similar challenges and where it is different, and highlighting what is needed to improve interprofessional education across different healthcare topics. In addition, by combining this framework with thematic analysis, we were able to balance structure with openness. The framework ensured that barriers and enablers were systematically categorized at the macro, meso, and micro levels, while the inductive thematic analysis allowed new domains to emerge directly from the data. This hybrid approach improved both the transparency and the richness of the findings, making it possible to capture context-specific insights without losing comparability across interprofessional education research.

That said, there are a few limitations to consider. First, participants were self-selected, both by attending a conference focused on interprofessional education and collaborative practice and by choosing to participate in a workshop on this topic. This introduces a risk of selection bias, as participants were likely already interested in, or supportive of interprofessional education, which may have influenced the type and direction of barriers and enablers identified. This may limit the generalizability of the findings. On the other hand, this self-selection may also be considered a strength. It likely reflects a stronger interprofessional identity, which serves as a source of motivation for interprofessional collaboration. Topics such as pharmacotherapy and medication safety appeared to act as identity triggers, simultaneously activating participants’ professional identity because they were perceived as highly relevant to their current or future professional roles. This, combined with a better understanding of their own healthcare system and cultural context, probably supported a more critical appraisal of the topics discussed during the World Café. A strong interprofessional identity, aligned with a congruent interprofessional frame of reference, enabled participants to identify deviations between preferred and actual practice, allowing them to generate a broad range of ideas while reflecting critically on gaps such as the lack of evaluation and evidence. Second, country representation was uneven, which largely reflects the fact that the World Café was conducted during a conference held in Qatar, where local attendance was higher. This composition was expected given the setting and allowed for in-depth discussion of context-specific challenges, while still incorporating perspectives from participants from other countries. Third, the guiding statements we used to steer the conversation may have influenced the direction of the discussions. However, this is an inherent feature of the World Café methodology, which uses predefined prompts to focus dialogue. While this may shape the scope of responses, it also helps ensure that discussions remain structured and comparable across groups. In addition, guiding statements can be beneficial because participants sometimes struggle to articulate their own opinions, which can result in low response rates [47]. The prompts therefore support participants in expressing their thoughts more clearly and encourage reflection on topics that might not have emerged spontaneously, enhancing the depth and richness of the discussion. Finally, no audio recordings were made, which may have limited the capture of detailed conversational nuance, the aim of the World Café was to generate and synthesize collective insights rather than to analyze individual discourse. The use of the post-it notes, iterative discussion rounds, the summaries of the moderators and participant validation supported the credibility and sufficiency of the data.

## Conclusion

This qualitative study used a World Café approach, conducted in an international conference setting, to explore barriers and enablers to implementing interprofessional education in pharmacotherapy and medication safety across macro-, meso-, and micro-levels according to the IECPCP framework [31, 33]. While many challenges, such as limited resources, institutional resistance, and unclear roles, were not specific to pharmacotherapy, the findings suggest that medication-related themes are particularly prominent at the macro level, where medication safety is shaped by broader factors such as prescribing legislation, professional rules, funding systems, and hierarchical traditions in healthcare. A key issue was the lack of robust outcome measures and evidence supporting interprofessional education in this context, which is essential for building support among policymakers and institutions.

These findings underscore the importance of cultural and professional context in relation to both professional and interprofessional identities. Context also provides a framework for understanding cultural differences and similarities, in this case in relation to pharmacotherapy and medication safety. These observations support the relevance of theoretical models such as the Extended Professional Identity Theory (EPIT), which suggests that interprofessional identity extends beyond, yet remains distinct from, professional identity and influences how learners perceive and evaluate interprofessional practice. Social identity, such as professional or interprofessional identity, is always context-based; yet identification remains a fundamental human tendency. It is primarily the contexts of different countries and cultures that create variation, shaping how these identities are experienced and expressed [18, 19].

Our results suggest that addressing cultural norms, clarifying professional roles, and improving evaluation strategies, which form the basis for the implementation recommendations proposed in this study, may contribute to activating a broader interprofessional identity, one in which future healthcare professionals value collaborative practice and share responsibility for safe prescribing. These factors can be understood as identity triggers, operating within overlapping educational and clinical contexts to influence which professional identities become most salient in practice. By engaging a diverse group of participants through a structured yet flexible methodology, this study provides new insights into the role of interprofessional education in pharmacotherapy and highlights the importance of embedding collaboration both at the educational and systemic levels.

### Recommendations (Fig. 2)

**Fig. 2:**
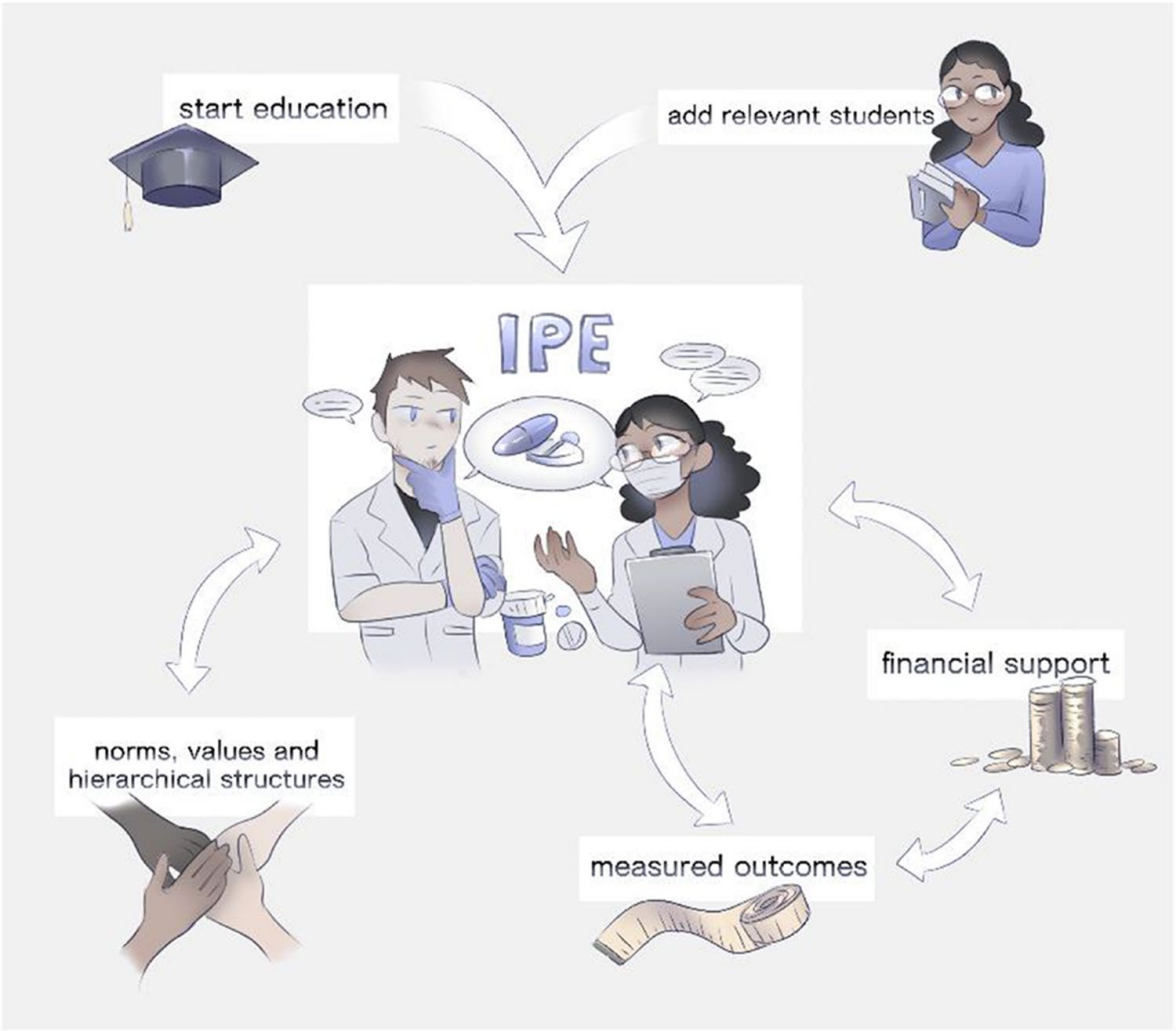
Key recommendations for interprofessional education in pharmacotherapy and medication safety. The illustration highlights how meaningful, practice-focused learning, involvement of relevant students, financial support, attention to norms and hierarchies, and measurable outcomes are interconnected in supporting effective interprofessional education implementation

Based on the barriers and enablers identified in this World Café study, the following practice-oriented recommendations may be considered when designing or implementing interprofessional education in pharmacotherapy and medication safety.

1. **Start with meaningful, practice-focused interprofessional education in medication safety**
  Create focused learning activities concerning challenges to medication safety, such as polypharmacy and safe prescribing. Engage students from relevant professions where their collaboration naturally fits. Start with small, real-life practical cases and build up to more teamwork-based sessions, for example an interprofessional medication review session for real-life patients.
2. **Use interprofessional education selectively for pharmacotherapy topics where collaboration matters most**
  Focus interprofessional learning on complex medication safety issues that require teamwork between prescribers, pharmacists, and nurses. This approach helps students strengthen both their professional expertise and interprofessional skills, for example during joint medication reviews or discussions of medication-related incidents.
3. **Move beyond measuring attitudes—assess real impact on medication safety and patient outcomes**
  To gain institutional and policy support, interprofessional pharmacotherapy and medication safety education should be evaluated by measuring improvements in clinical practice and patient safety, for example by tracking reductions in medication errors and measuring patient satisfaction.
4. **Start implementing interprofessional education even if cultural barriers remain—culture changes with experience**
  Do not wait for hierarchies or cultural barriers in pharmacotherapy to disappear before starting. Experience shows that these barriers begin to shift once interprofessional education is embedded in everyday education and practice. For example with small interprofessional prescribing exercises with students from different professions, such as medical and nursing students, where hierarchical dynamics exist. These exercises encourage reflection on role negotiation and collaboration within real-world prescribing contexts.

## Supplementary Information

Below is the link to the electronic supplementary material.

Supplementary file1 (PDF 213 KB)

Supplementary file2 (PDF 219 KB)

Supplementary file3 (PDF 216 KB)

## Data Availability

No datasets were generated or analysed during the current study.
