# Supplementary material for: Cross-cultural and international barriers and enablers to medication safety and pharmacotherapy: insights from a World Café study on interprofessional education
Source: Eur J Clin Pharmacol. 2026 Jul 10;82(8):198. doi: 10.1007/s00228-026-04128-1 (PMC13350127; doi:10.1007/s00228-026-04128-1)
Supplement: Supplementary file 1 — Supplementary file1 (PDF 213 KB) [file 228_2026_4128_MOESM1_ESM.pdf]

Theme of barrier	Barriers	Enabler
Culture and attitude	A culture of transparency to discuss medication side effects and/or errors	Promoting open communication
		Encouraging open discussions on medication safety to normalize transparency
	Professional overconfidence hinders interprofessional collaboration	Using safety discussions to challenge overconfidence
	A false sense of safety hinders the need for interprofessional education	Encouraging open discussions on medication safety to challenge false security
		Using quality improvement as a framework to promote reflection
	Cultural hierarchies hinder interprofessional collaboration	Embedding interprofessional collaboration as an accreditation requirement
		Implementing internationally aligned curricula to encourage openness
Policy and governance	Lack of motivation and urgency among stakeholders hinders the integration	Communicating a clear sense of urgency among stakeholders
		Leveraging quality improvement initiatives to drive engagement
		Raising awareness of the value of interprofessional education
		Increasing student awareness to create bottom-up momentum
	Lack of perceived urgency among policymakers delays implementation	Framing interprofessional education as an urgent healthcare priority
		Showcasing policy precedents where interprofessional education is already embedded
		Raising awareness of the value of interprofessional education
		Demonstrating how interprofessional education supports system-wide improvements
Resources and support	High costs associated with implementation	Conduct cost-effectiveness studies
Evaluation and evidence	Lack of clear patient outcome data	Define both learning and health outcomes
	No standard definitions of outcomes for research	Create shared definitions and goals
	Difficulty linking interprofessional education to measurable impact	Use standardized materials and case studies
		Focus on healthcare-specific outcomes
		Include retrospective or short-term studies
	Limited research and evidence base for interprofessional education	Build awareness of the importance of interprofessional education
	Challenges in long-term impact measurement	
	Too many confounding factors that can affect the association	

Table S1. Macro level: thematic barriers and enablers. Overview of barriers and enablers at the macro level, according to the IECPCP framework, focusing on systemic and policy-related factors such as regulations, funding, and professional hierarchies.
