# Supplementary material for: Cross-cultural and international barriers and enablers to medication safety and pharmacotherapy: insights from a World Café study on interprofessional education
Source: Eur J Clin Pharmacol. 2026 Jul 10;82(8):198. doi: 10.1007/s00228-026-04128-1 (PMC13350127; doi:10.1007/s00228-026-04128-1)
Supplement: Supplementary file 2 — Supplementary file2 (PDF 219 KB) [file 228_2026_4128_MOESM2_ESM.pdf]

Theme of barrier	Barriers	Enabler
Roles and identity	Language and communication barriers between professions hinder effective collaboration	Using standardized medical terminology
		Improving communication skills by training
	Professional ego and reluctance to adapt to new systems limit engagement with interprofessional approaches	Promoting visionary leadership
		Engaged and supportive stakeholders
		Offering role models and mentors
		Building a supportive team culture
Structure and curriculum	Inflexible teaching mindsets and lack of autonomy hinder the implementation of interprofessional medication safety education	Giving educators time and freedom to shape the curriculum
		Providing clear policies and guidance for interprofessional education
		Offering training by facilitators who believe in interprofessional education
		Using practical examples and case-based materials
		Ensuring mentors are available to support teachers
	Limited curriculum time and space restrict the integration of interprofessional education	Integrating interprofessional education into core curriculum
		Providing protected time and space for interprofessional education
		Making interprofessional education easy to implement in new programs
		Ensuring low-resource solutions are available
		Building flexibility into the curriculum structure
	Institutional curriculum design often prioritizes certain areas, leaving others underdeveloped	Embedding interprofessional education in all health professional programs
		Encouraging collaboration between teachers and practitioners
	Poor coordination between different programs' timetables obstructs joint learning opportunities	Designing curriculum to allow shared learning moments
		Providing flexibility for cross-program collaboration
	Bias in curricula toward single-discipline perspectives limits interprofessional exposure	Integration into national curriculum guidelines
		Making interprofessional education compulsory in all healthcare programs
		Embedding interprofessional education throughout all disciplines
Policy and governance	Lack of sustainable implementation processes reduces long-term impact of interprofessional initiatives	Establishing clear escalation and support processes
		Providing consistent guidelines and structures
	Competing priorities within institutions prevent focus on interprofessional education	Gaining strong support from management
		Emphasizing the added value of interprofessional education in healthcare shortages

		Securing commitment from all stakeholders
	Lack of institutional and environmental support discourages implementation efforts	Developing a culture that supports interprofessional learning
		Strengthening leadership at the organizational level
	Sudden changes and last-minute decisions disrupt structured implementation	
Resources and support	Interprofessional education is perceived as a poor financial investment or a waste of resources	Providing dedicated funding from the university
		Showing the long-term value through improved safety outcomes
		Offering financial incentives to support interprofessional education
	Lack of financial, human, and infrastructural resources from institutions hampers development	Securing university-level funding
		Training and assigning enough facilitators
		Using low-cost and scalable interprofessional education formats
	Funding is misdirected toward less impactful initiatives, leaving interprofessional education underfunded	Offering financial incentives aligned with interprofessional education goals
		Redirecting funding toward interprofessional education development and delivery
Culture and attitude	Strong resistance to change slows the adoption of interprofessional practices	Promoting visionary leadership
		Highlighting real incidents (e.g. medical errors) as learning points
		Using passionate facilitators to lead change
		Showing how interprofessional education improves patient safety in practice
Evaluation and evidence	Difficulty designing and validating assessments	Set clear and focused study goals
	Limited understanding of complex issues, like polypharmacy, reduces perceived relevance of interprofessional education	Using real-world cases and examples
		Highlighting risks through data on medication errors
		Integrating interprofessional education in clinical settings to improve understanding

Table S2. Meso level: thematic barriers and enablers. Overview of barriers and enablers at the meso level, according to the IECPCP framework, covering organizational and curricular factors including leadership, resources, and institutional structures.
