# Supplementary material for: Cross-cultural and international barriers and enablers to medication safety and pharmacotherapy: insights from a World Café study on interprofessional education
Source: Eur J Clin Pharmacol. 2026 Jul 10;82(8):198. doi: 10.1007/s00228-026-04128-1 (PMC13350127; doi:10.1007/s00228-026-04128-1)
Supplement: Supplementary file 3 — Supplementary file3 (PDF 216 KB) [file 228_2026_4128_MOESM3_ESM.pdf]

Theme of barrier	Barriers	Enabler
Resources and support	Rushed collaboration and lack of time hinder meaningful interprofessional interaction	Integrating interprofessional education into problem based learning/team based learning without adding extra time
		Encouraging shared activities during clinical education
		Building in reflection moments to slow down and debrief
	Lack of experts limits the development and delivery of interprofessional education	Offering faculty development training
		Improving quality of training materials and facilitators
		Involving professionals from each discipline in teaching teams
Structure and curriculum	Uncertainty about how to translate interprofessional education into clear competencies complicates curriculum integration	Including interprofessional education in problem based learning/team based learning cases with defined outcomes
		Providing training on how to frame interprofessional education into competencies
		Making evaluation part of implementation
	Interprofessional education interventions may not feel authentic to learners	Align interprofessional education with real professional experiences
	Implementation of interprofessional education competencies lacks structure and guidance	Developing faculty training that supports structured interprofessional education implementation
		Using team collaboration from multiple schools to co-design content
		Including interprofessional education in routine educational activities (e.g., vignettes)
Roles and identity	Strong professional identity can cause discomfort or shame when stepping outside traditional roles	Encouraging contribution awareness and role value within teams
		Building awareness of professional identity as part of collaboration
		Promoting peer-led teaching to shift perspectives (students become teachers)
	Lack of role clarity undermines effective interprofessional collaboration	Training students to understand team roles and daily routines
		Using mixed professional teams to define and discuss roles
		Raising awareness of each profession's contribution through case examples
Culture and attitude	Fear of interpersonal conflict discourages open communication between professions	Creating safe learning environments through structured facilitation
		Providing training in team communication and feedback
		Using positive interprofessional education experiences to build confidence
	Lack of accountability reduces responsibility for contributing to interprofessional efforts	Making interprofessional education evaluation part of student and teacher responsibilities
		Highlighting the importance of individual contributions to patient safety
		Creating shared ownership through interprofessional project work

	Resistance from some professionals limits full participation in interprofessional education activities	Using positive experiences to overcome resistance
		Emphasizing the need to prevent incidents through collaboration
		Building awareness of real-life complications to show urgency
		Using behavior-focused faculty development training
		Ensuring teaching about the importance of interprofessional education
		Awareness of further complications

Table S3. Micro level: thematic barriers and enablers. Overview of barriers and enablers at the micro level, according to the IECPCP framework, addressing individual learners and educators, their skills, attitudes, and direct teaching and learning interactions.
